# Prescription of Potentially Inappropriate Medication in Older Inpatients of an Internal Medicine Ward: Concordance and Overlap Among the EU(7)-PIM List and Beers and STOPP Criteria

**DOI:** 10.3389/fphar.2021.676020

**Published:** 2021-07-30

**Authors:** Carla Perpétuo, Ana I. Plácido, Daniela Rodrigues, Jorge Aperta, Maria Piñeiro-Lamas, Adolfo Figueiras, Maria Teresa Herdeiro, Fátima Roque

**Affiliations:** ^1^ Health Sciences School, Polytechnic Institute of Guarda, Guarda, Portugal; ^2^ Local Health Unit of Guarda, Guarda, Portugal; ^3^ Research Unit for Inland Development, Polytechnic Institute of Guarda (UDI/IPG), Guarda, Portugal; ^4^ Consortium for Biomedical Research in Epidemiology and Public Health (CIBER en Epidemiología y Salud Pública- CIBERESP), Santiago de Compostela, Spain; ^5^ Health Research Institute of Santiago de Compostela (IDIS), Santiago de Compostela, Spain; ^6^ Department of Preventive Medicine and Public Health, University of Santiago de Compostela, Santiago de Compostela, Spain; ^7^ Department of Medical Sciences, Institute of Biomedicine (iBiMED-UA), University of Aveiro, Aveiro, Portugal; ^8^ Health Science Research Center (CICS/UBI), University of Beira Interior, Covilhã, Portugal

**Keywords:** potentially inappropriate medication, internal medicine ward, older adults, EU(7)-PIM list, AGS 2019 Beers criteria, STOPP v2 criteria

## Abstract

**Background:** Age-related comorbidities prone older adults to polypharmacy and to an increased risk of potentially inappropriate medication (PIM) use. This work aims to analyze the concordance and overlap among the EU(7)-PIM list, 2019 Beers criteria, and Screening Tool of Older Person’s Prescriptions (STOPP) version 2 criteria and also to analyze the prevalence of PIM.

**Methods:** A retrospective cohort study was conducted on older inpatients of an internal medicine ward. Demographic, clinical, and pharmacological data were collected, during March 2020. After PIM identification by the EU(7)-PIM list, Beers criteria, and STOPP v2 criteria, the concordance and overlap between criteria were analyzed. A descriptive analysis was performed, and all the results with a *p*-value lower than 0.05 were considered statistically significant.

**Results:** A total of 616 older patients were included in the study whose median age was 85 (Q1–Q3) (78–89) years. Most of the older patients were male (51.6%), and the median (Q1–Q3) number of days of hospitalization was 17 (13–22) days. According to the EU(7)-PIM list, Beers criteria, and STOPP criteria, 79.7, 92.0, and 76.5% of older adults, respectively, used at least one PIM. A poor concordance (<63.4%) among criteria was observed. An association between PIM and the number of prescribed medicines was found in all applied criteria. Moreover, an association between the number of PIMs and diagnoses of endocrine, nutritional, and metabolic diseases, mental, behavioral, and neurodevelopmental disorders, and circulatory system diseases and days of hospitalization was observed according to Beers criteria, and that with diseases of the circulatory system and musculoskeletal system and connective tissue was observed according to STOPP criteria.

**Conclusion:** Despite the poor concordance between the EU(7)-PIM list, 2019 Beers, and STOPP v2 criteria, this work highlights the need for more studies in inpatients to develop strategies to facilitate the identification of PIM to decrease the high prevalence of PIM in hospitalized patients. The poor concordance among criteria also highlights the need to develop new tools adapting the existing criteria to medical ward inpatients.

## Introduction

Age-related pharmacokinetic and pharmacodynamics changes cause a decrease in the ability to adapt to external environment alterations, increased susceptibility to the disease, a lesser capacity to recovery that causes a modified response to medications, greater susceptibility to the occurrence of adverse drug reactions (ADRs) (Alvis and Hughes, 2015; Gutierrez Valencia et al., 2016; Giardina et al., 2018), and an upsurge need for health resources (Stegemann et al., 2010).

Polypharmacy, the use of five or more medicines (Lee et al., 2020), is quite common in patients with multiple comorbidities and is considered a factor for functional decline in older adults, which increases the chance of medication-related problems (Garcia-Caballero et al., 2018; Lee et al., 2020). Overall, polypharmacy is associated with increased consumption of potentially inappropriate medication (PIM) (Oktora et al., 2020).

In this context, medicines are considered appropriate for older adults, when there is a clear, evidence-based indication that these medicines are generally well tolerated and have a favorable benefit/risk ratio in older adults (Laroche et al., 2019).

PIMs are medicines in which the potential risk of occurrence of ADR may be greater than the clinical benefit (Renom-Guiteras et al., 2015) that can be driven from their use, particularly when there is scientific evidence of alternatives that may be safer, so it becomes essential to optimize the prescription of medicines in aged population (Renom-Guiteras et al., 2015; Grina and Briedis, 2017). Several tools using explicit or implicit criteria have been developed to allow the identification of PIM and prevent PIM-associated negative outcomes (Chang and Chan, 2010; Kaufmann et al., 2014; Lucchetti and Lucchetti, 2017; Motter et al., 2018). Because older inpatients are at particular risk of PIM (Sinvani et al., 2013; Nothelle et al., 2017), it is fundamental to understand what drives the use of PIM in hospitals to design interventions to restraint PIM use in this setting. According to our knowledge, the overlap and concordance among criteria remain poorly reported in all settings. Therefore, we sought to analyze the concordance and overlap between the EU(7)-PIM list, 2019 Beers criteria, and Screening Tool of Older Person’s Prescriptions (STOPP) version 2 (v2) criteria in the identification of PIM in older adult inpatients in a general internal medicine ward. Also, the prevalence of PIM, using the EU(7)-PIM list, Beers criteria, and STOPP criteria, will be analyzed.

## Materials and Methods

### Source of Data and Study Population

A retrospective cohort study was performed to examine the overlap and concordance between the EU(7)-PIM list, 2019 American Geriatric Society (AGS) Beers criteria, and STOPP v2 criteria on the detection of PIM among older inpatients of an internal medicine ward of a first-level hospital belonging to the NUTS II (Nomenclatura das Unidades Territoriais para Fins Estatísticos/Nomenclature of Territorial Units for Statistics) area of Portugal defined by the Regional Administration of Health Center (Administração Regional de Saúde do Centro/ARS-C). The hospital where the study takes place covers a total of 51243 older adults (PORDATA, 2020) and has a total of 68 beds in the general internal medicine ward.

Eligible to participate in the study were all older patients (aged ≥65) admitted in the internal medicine ward during 2019 and hospitalized for at least 4 days, during 2019. Data were encoded and retrospectively collected, during March 2020, from the hospital’s electronic medical record and included patient age, patient gender (male/female), patient diagnoses, hospitalization days, drugs prescribed, and also medical and laboratory tests. This study obtained the ethical approval of the hospital ethical committee and authorization from the hospital board (ref. 01167) on February 7, 2020. All data were retrospectively encoded without any possibility of identification and were treated according to the European Union (EU) General Data Protection Regulation (GDPR).

### Data Collection

All drugs prescribed to older patients during the study period were analyzed and PIM identified by the three tools used by two independent researchers (CP and DR), and any disagreement regarding PIM classification was resolved by a third researcher (FR) (Supplementary Tables 1–10).

PIM detection tools used were as follows:

a) The EU(7)-PIM list was developed through the consensus of experts from seven European countries: Estonia, Finland, France, Germany, Holland, Spain, and Sweden (Renom-Guiteras et al., 2015). The purpose of this list is to enable the identification and comparison of PIM prescription profiles for the elderly across the European community. This list comprises 275 active substances, 7 classes of drugs, belonging to 55 therapeutic classes, and 34 pharmacotherapeutic groups. In this work, the list adapted to Portuguese reality was used (Rslbodrigues et al., 2020).
b) 2019 AGS Beers criteria (American Geriatrics Society Beers Criteria® Update Expert, 2019) developed in the United States are one of the most used tools and use explicit criteria. This tool has already undergone several revisions, the last being 2019, and includes six tables: table 2 listing “potentially inappropriate medications in older patients apart from the clinical condition,” table 3 “medication use in older adults due to drug–disease or drug–syndrome interactions that may exacerbate the disease or syndrome,” table 4 “potentially inappropriate medications in older patients considering the clinical condition,” table 5 listing “potentially inappropriate medications—drugs to be used with caution in older adults,” table 6 listing “potentially clinically important drug–drug interactions that should be avoided in older adults,” and table 7 listing “medications that should be avoided or have their dosage reduced with varying levels of kidney function in older adults” (American Geriatrics Society Beers Criteria Update Expert, 2019).
c) STOPP v2 criteria (O’mahony et al., 2015). The STOPP/START criteria were created in 2008 and also emerged as an European response to drug-related problems (DRPs), to identify whether the medical prescription is suitable for older adults (O’mahony et al., 2015). The list was revised in 2015 and is organized by physiological systems. The STOPP/START tool includes 114 criteria: 80 STOPP criteria and 34 START criteria (O’mahony et al., 2015).

Drugs were classified according to the Anatomical and Therapeutic Chemical Classification (WHO, 2021), and patients’ diagnoses were classified according to the International Statistical Classification of Diseases and Related Health Problems (ICD-10 Second Edition).

### Statistical Analysis

Numerical and ordinal data were presented in frequency and percentage and using mean, median, percentile 25 and percentile 75, and standard error. A comparative analysis was performed between the results obtained for the three PIM identification tools, and the agreement between them was determined through the Lin coefficient. The prevalence of PIM was defined as the number of patients taking at least one PIM and was calculated using 95% CI. A medicine was considered a PIM if it is identified for at least one tool.

The pro package from the statistical software R was used to estimate the sample size. The sample size was computed using an estimated prevalence of 50% with a margin error of 4%. To ensure the precision of the data, the program reported that the sample should have at least 601 patients. This study included all the 616 patients that have been admitted to the internal medicine ward of the hospital, during 2019.

The free statistical software R (v4.0.0) was used to perform statistical analysis. A generalized linear model was developed for the dependent variables. Bivariate analysis was performed to select independent variables with a *p*-value < 0.2. The selected variables were studied in multivariate analysis, and those that had greater statistical significance were successively eliminated, on the condition that the coefficients of the main exposure variables did not change by more than 10% and that Schwarz’s Bayesian Information Criterion (BIC) improved. Considering hospitalization days as a dependent variable, a risk analysis was performed using Cox regression.

To correlate PIM identified with the multiple diagnoses of the patients, the total number of diagnoses per patient was added to the generalized linear model as the Independent variable and the number of PIMs identified as the dependent variable. The model was adjusted according to the sex and age of the patients.

## Results

### Study Population Characteristics

During the study period, 662 older patients were admitted to the internal medicine ward. Of these, 46 were excluded from the study because hospitalization was less than 4 days. Table 1 shows the characteristics of the 616 older patients included in the study. The median (Q1–Q3) age was 85.00 (78–89) years, and 48.16% of the participants were female. The median (Q1–Q3) number of hospitalization days was 12.00 (8–20), and the median (Q1–Q3) number of medicines taken per patient during the hospitalization period was 17.00 (13–22). Of the total number of older people included in the study, 547 (88.7%) were discharged from the hospital, 13 (2.1%) were transferred from another ward or another hospital, and 67 patients (9.1%) died.

**TABLE 1:** Study population characteristics.

Study population Characteristics N (%)	Participants N = 616
Age (years)	—
Median (Q1–Q3)	85.00 (78.0–89.0)
65–74	98 (15.90%)
75–84	206 (33.40%)
≥85	312 (50.70%)
Sex	—
Female	298 (48.40%)
Male	318 (51.60%)
Hospitalization days	—
Median (Q1–Q3)	12 (8–20)
Range (minimum and maximum)	4–90
No. of prescribed drugs	—
Median (Q1–Q3)	17 (13–22)
Range (minimum and maximum)	4–50
ICD-10 diagnostics	N = 3,873
A00-B99, certain infectious and parasitic diseases	96 (2.50%)
C00-D49, neoplasms	79 (2.00%)
D50-D89, diseases of the blood and blood-forming organs and certain disorders involving the immune mechanism	220 (5.70%)
E00-E89, endocrine, nutritional, and metabolic diseases	636 (16.40%)
F0-F99, mental, behavioral, and neurodevelopmental disorders	140 (3.60%)
G00-G99, diseases of the nervous system	82 (2.10%)
H00-H59, diseases of the eye and adnexa	11 (0.30%)
H60-H95, diseases of the ear and mastoid process	14 (0.40%)
I00-I99, diseases of the circulatory system	829 (21.40%)
J00-J99, diseases of the respiratory system	415 (10.70%)
K00-K95, diseases of the digestive system	125 (3.20%)
L00-L99, diseases of the skin and subcutaneous tissue	50 (1.30%)
M00-M99, diseases of the musculoskeletal system and connective tissue	80 (2.10%)
N00-N99, diseases of the genitourinary system	396 (10.20%)
Q00-Q99, congenital malformations, deformations, and chromosomal abnormalities	1 (0.00%)
R00-R99, symptoms, signs, and abnormal clinical and laboratory findings, not elsewhere classified	278 (7.20%)
S00-T88, injury, poisoning, and certain other consequences of external causes	53 (1.40%)
V00-Y99, external causes of morbidity	32 (0.8%)
Z00-Z99, factors influencing health status and contact with health services	336 (8.70%)

Q1- percentile 25, Q3-percentile 75

A total of 3,873 diagnoses were registered for all the included patients. 21.4% of the diagnoses belong to the group of diseases related to the circulatory system, 16.4% to endocrine, nutritional, and metabolic diseases, and 10.7% to respiratory system diseases (Table 1).

### Prevalence of PIM According to the EU(7)-PIM List and Beers and STOPP Criteria

Of 11159 prescribed medicines (mean per patient 18.12 ± 7.33), 285 were different active substances and were analyzed using the EU(7)-PIM list and Beers and STOPP criteria to evaluate the prevalence of PIM (Table 2 and Table 3)**.**

**TABLE 2:** Number of PIMs identified in our sample according to the EU(7)-PIM list and Beers and STOPP criteria.

Frequency of PIMs	Tool		
	EU(7)-PIM list	Beers criteria	STOPP criteria
	N (PCT; 95% CI)	N (PCT; 95% CI)	N (PCT; 95% CI)
0	125 (20.30; 0.17–0.24)	37 (6.0; 0.04–0.08)	145 (23.50; 0,20–0.27)
1	153 (24.80; 0.22–9.29)	106 (17.20; 0.14–0.20)	165 (26.80; 0.23–0.31)
2	163 (26.50; 0.23–0.30)	152 (24.70; 0.21–0.28)	130 (21.10; 0.18–0.25)
3	76 (12.4; 0.10–0.15)	126 (20.50; 0.17–0.24)	88 (14.30; 0.12–0.17)
4	52 (8.40; 0.06–0.11)	76 (12.30; 0.10–0.15)	37 (6.90; 0.04–0.08)
≥5	46 (7.50; 0.06–0.10)	119 (19.30; 0.16–0.23)	51 (8.30; 0.06–0.11)

**TABLE 3:** The five most consumed PIMs according to the EU(7)-PIM list and Beers and STOPP criteria.

Position	EU(7)-PIM list	*n*	% PIM	Beers 2019	*n*	% PIM	STOPP v2	*n*	% PIM
1	Metoclopramide	192	a) 16.75%	Furosemide	437	a) 23.90%	Haloperidol	148	a) 12.80%
			b) 31.20%			b) 71%			b) 24%
2	Haloperidol	143	a) 12.10%	Metoclopramide	192	a)10.50%	Quetiapine	88	a) 7.60%
			b) 23.20%			b) 31.20%			b) 14.30%
3	Bisacodyl	110	a) 10.4%	Haloperidol	148	a) 8.10%	Spironolactone	79	a) 6.80%
			b) 20%			b) 24.00%			b) 12.80%
4	Alprazolam	58	a) 4.90%	Spironolactone	107	a) 5.90%	Lorazepam	69	a) 6.00%
			b) 9.40%			b) 17.40%			b) 11.2%
5	Digoxin	57	a) 4.80%	Quetiapine	88	a) 4.80%	Oxazepam	65	a) 5.60%
			b) 9.20%			b) 14.30%			b) 10.50%

a) percentage of PIMs per tool; b) percentage of PIMs per patient (N = 616).

According to the EU(7)-PIM list adapted to Portuguese reality, 63 of the analyzed medicines were considered PIM, with a total of 1,146 PIMs detected in our sample. The median (Q1–Q3) number of PIMs per patient was 2 (1–3). It was also observed that 79.70% of the participants take at least one PIM (Table 2). The maximum number of PIMs per patient detected was 10, consumed by one patient (0.20%). The majority of the patients (51.30%) take one or two PIMs. Overall, the most consumed PIMs according to the EU(7)-PIM list were metoclopramide, haloperidol, and bisacodyl consumed by 31.2, 23.2, and 17.9% of our sample, respectively, representing a total of 38.9% of the PIMs identified by the EU(7)-PIM list (Table 3; Supplementary Table 1).

According to Beers criteria, considering 77 analyzed medicines, we have identified a total of 1,829 PIMs. It was also observed that 94.00% of the patients take at least one PIM, 17.20% of the participants take one PIM, and 0.20% take thirteen PIMs. Most of the patients (62.40%) take more than one and less than four PIMs (Table 2). The median (Q1–Q3) number of PIMs per patient observed was 3 (2–4). Furosemide, metoclopramide, and haloperidol were the most consumed PIMs, used by 71.0, 31.20, and 24.00% of the inpatients, respectively, representing a total of 42.50% of the PIMs detected by this tool (Table 3; Supplementary Table 2).

According to table 2 of the Beers criteria (“potentially inappropriate medications in older patients apart from the clinical condition”), the participants consumed a total of 979 PIMs (Supplementary Table 3), with metoclopramide and haloperidol being the most consumed, taken by 192 and 148 participants, respectively. The application of Table 3 (“potentially inappropriate medications in older patients considering the clinical condition”) of the Beers criteria detected a total of 221 PIMs (Supplementary Table 4). The application of table 4 of Beers criteria (“potentially inappropriate medications—drugs to be used with caution in older adults”) allows the detection of 1,226 drugs that should be used with caution in older adults (Supplementary Table 5). The application of table 5 of Beers criteria (potentially clinically important drug–drug interactions that should be avoided in older adults) identified 263 potential drug–drug interactions that should be avoided in older patients (Supplementary Table 6). The application of table 6 of Beers criteria (medications that should be avoided or have their dosage reduced with varying levels of kidney function in older adults) revealed the presence of six PIMs (Supplementary Table 7). The frequency of anticholinergic drugs was 133 (table 7 of Beers criteria) (Supplementary Table 8).

It was possible to apply 40 specific STOPP criteria to the prescribed medication, obtaining a total of 1156 PIMs. According to this tool, 76.50% of our sample consume at least one PIM, 26.80% of the sample consume one PIM, 8.30% of the sample consume five or more PIMs, and 0.50% consume ten PIMs (Table 3). The median (Q1–Q3) number of PIMs per participant was 2 (1–3). The section of the STOPP criteria where the highest number of PIMs was obtained was section K, which refers to drugs that predictably increase the risk of falls in elderly people by 42.3%. The amount of PIMs obtained when applying each of the criteria is greater than the amount of PIMs found in table 6 (1,156 PIMs) since several drugs can be PIMs due to multiple criteria (Supplementary Table 9).

### Concordance and Overlap Among the EU(7)-PIM List, Beers Criteria, and STOPP Criteria

After the analysis of PIM by each tool, we observed that, according to the EU(7)-PIM list and Beers criteria, metoclopramide should be used with caution in older adults (EU(7)-PIM list) and is considered a PIM in older adults, and apart from the clinical condition (Beers criteria), this drug was considered a PIM in all patients that use it. According to STOPP criteria, metoclopramide can exacerbate Parkinsonian symptoms, in patients with Parkinson disease, so this drug is a PIM in 13 patients.

Haloperidol was the most prevalent PIM identified by the STOPP criteria, the second most prevalent according to the EU(7)-PIM list, and the third most observed according to Beers criteria. In the 148 patients that take haloperidol, 143 use a single dose superior to 2 mg or take more than 5 mg/d, and for these reasons, it was considered a PIM according to the EU(7)-PIM list. According to STOPP criteria, this drug predictably increases the risk of falls in older people (may cause gait dyspraxia, Parkinsonism), and according to the Beers criteria, haloperidol should be avoided in older adults, and this is the main reason for considering haloperidol a PIM in all patients that use it.

Bisacodyl was one of the most prevalent PIMs identified by the EU(7)-PIM list; due to the duration of treatment (>3 days), according to Beers and STOPP criteria, this drug is not a PIM. All applied criteria considered alprazolam a PIM, and the common reason according to the tools used is because this drug should be avoidable in older adults independently of their clinical condition. According to Beers criteria, furosemide should be avoided in older adults and is considered a PIM in all patients that use it. According to STOPP criteria, furosemide is a loop diuretic for dependent ankle edema without clinical, biochemical, or radiological evidence of heart and liver failure, nephrotic syndrome, or renal failure (leg elevation and/or compression hosiery usually being more appropriate) and may exacerbate incontinence.

Therefore, spironolactone being considered a PIM by all applied criteria, the reasons and the number of patients with this PIM are divergent. According to the EU(7)-PIM list of the 107 patients that use spironolactone, only 23 use a dose more than 25 mg/day (the reason for PIM); according to Beers criteria, this drug should be avoided in older adults, so it is considered a PIM in all patients (107) that use it. STOPP criteria considered that 79 patients use spironolactone as a PIM because in these patients’ serum, potassium was not regularly monitored. Beers and STOPP criteria considered quetiapine as a PIM in all patients that use it because this drug should be avoided in older adults (Beers criteria) and predictably increases the risk of falls in older people (STOPP criteria); according to the EU(7)-PIM list, this drug is not a PIM. Lorazepam is a PIM by all applied criteria, according to the EU(7)-PIM list in 31 of the 69 patients that use it due to the high dose (>1 mg/d). According to Beers criteria, this drug should be avoided in older adults and is considered a PIM in all patients (69) that use it. According to the STOPP criteria, lorazepam is a PIM in all patients because it may cause reduced sensorium and, in patients with acute or chronic respiratory failure, there is a risk of exacerbation of respiratory failure.

**FIGURE 1:**
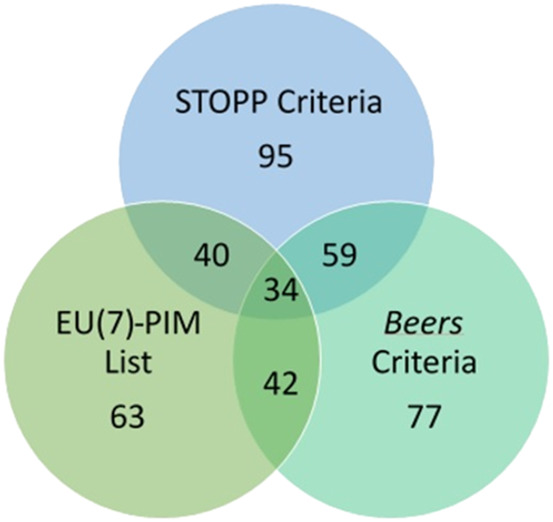
PIM identified by the EU(7)-PIM list, AGS 2019 Beers criteria, and STOPP version 2 criteria.

Considering the three PIM classification tools applied, the EU(7)-PIM list has 42 PIMs in common with the 2019 Beers criteria and 40 PIMs in common with version 2 of the STOPP criteria, whereas the 2019 Beers criteria have 59 PIMs in common with version 2 of the STOPP criteria. The three tools have in common 34 drugs (Figure 1).

### PIM-Associated Therapeutic Groups According to the Applied Criteria

To better understand the concordance between the different tools, PIMs identified by each tool were grouped according to the anatomical group (Table 4), and it was observed that, of the 1,901 prescribed medicines belonging to the alimentary tract and metabolism, 19.52% were considered PIM according to the EU(7)-PIM list, 11.00% were PIM according to Beers criteria, and only 1.84% were classified as PIM by the STOPP criteria. The analysis of the 2,283 medicines belonging to the cardiovascular system group by the EU(7)-PIM list, Beers criteria, and STOPP criteria revealed that 9.33, 29.04, and 12.57%, respectively, are PIM.

**TABLE 4:** Prevalence of PIM identified in our sample according to the pharmacological group.

Pharmacological groups (1° level anatomical group)	Tool			
	Prescribed medicine (N)	EU(7)-PIM list	Beers criteria	STOPP criteria
		N (PCT; 95% CI)	N (PCT; 95% CI)	N (PCT; 95% CI)
A-alimentary tract and metabolism	1901	371 (19.52%; 0.18–0.21)	209 (11.00%; 0.01–0.12)	35 (1.84%; 0.01–0.03)
B-blood and blood-forming organs	2,606	49 (1.88%; 0.014–0.02)	76 (2.92%; 0.02–0.04)	24 (0.92%; 0.01–0.01
C-cardiovascular system	2,283	213 (9.33%; 0.08–0.11)	663 (29.04%; 0.27–0.31)	287 (12.57%; 0.11–0.14)
D-dermatologicals	28	0	0	0
G-genitourinary system and sex hormones	144	2 (1.39%; 0.00–0.05)	0	2 (1.39%; 0.00–0.05)
H-systemic hormonal preparations, except sex hormones and insulins	220	0	2 (0.9%; 0.00–0.03)	25 (11.36; 0.07–0.16)
J-anti-infective for systemic use	1,043	2 (0.19%; 0.00–0.01)	30 (2.88; 0.02–0.04)	0
L-antineoplastic and immunomodulating agents	17	0	0	0
M-musculo-skeletal system	151	37 (24.50%; 0.18–0.32)	39 (25.83; 0.19–0.34)	17 (11.26%; 0.07–0.17)
N-nervous system	1913	464 (24.26%; 0.22–0.26)	802 (41.92%; 0.40–0.44)	753 (39.36%; 0.37–0.42)
P-antiparasitic products, insecticides, and repellents	2	0	0	0
R-respiratory system	800	8 (1%; 0.00–0.02)	8 (1%; 0.00–0.02)	13 (1.63%; 0.01–0.02)
S-sensory organs	27	0	0	0
V-various	24	0	0	0

According to the STOPP criteria, 11.36% of the 220 prescribed medicines of the group systemic hormonal preparations, except sex hormones and insulins, were PIM; according to the Beers criteria, only 0.9% of these groups of medicines are PIM; and according to the EU(7)-PIM list, none of them are PIM. Regarding the medicines from the musculoskeletal system group, 24.50% were PIM according to the EU(7)-PIM list, 25.83% were PIM according to the Beers criteria, and only 11.26% were PIM according to the STOPP criteria. We also observed that, of the 1,913 medicines belonging to the nervous system group, 24.26% were PIM according to the EU(7)-PIM list, 41.92% were PIM according to the Beers criteria, and 39.36% were PIM according to the STOPP criteria. According to the EU(7)-PIM list, the most prescribed PIM pharmacotherapeutic groups are the musculoskeletal system (24.50%), nervous system (24.26%), and alimentary tract and metabolism (19.56%). The most frequent PIM, according to Beers criteria, belongs to the nervous system group. According to the STOPP criteria, the most frequent PIM belongs to the pharmacotherapeutic groups—nervous system (39.36%), cardiovascular system (12.57%), and systemic hormonal preparations, except sex hormones and insulins (11.36%).

To analyze the agreement between the three criteria, we used Lin’s concordance correlation coefficient and observed a poor concordance between criteria (Table 5).

**TABLE 5:** LIN concordance correlation coefficient.

PIM tool	CCC (95% CI)
*EU(7)-PIM list* vs. STOPP	0.581 (0.521–0.635)
*EU(7)-PIM list* vs. *Beers*	0.596 (0.549–0.640)
STOPP vs. *Beers*	0.633 (0.583–0.678)

### PIM-Associated Factors

An association between the PIM detected through the application of the Beers criteria and patients with diagnoses of endocrine, nutritional, and metabolic diseases (ICD-10; E00-E89), mental, behavioral, and neurodevelopmental disorders (ICD-10; F01-F99), and circulatory system diseases (ICD-10; I00-I99) was observed (Table 6). PIMs detected by STOPP criteria are associated with patients diagnosed with diseases of the circulatory system (ICD-10; I00-I99) and with diseases of the musculoskeletal system and connective tissue (ICD-10; M00-M99). It was observed that the variable days of hospitalization only obtained statistical significance in relation to the PIM obtained with the application of the Beers criteria. The impact of the number of diagnoses on the effect of PIM is found to be small (OR∼1) and statistically significant (Table 6).

**TABLE 6:** Factors associated with PIM prevalence.

PIM tool	Variable	Adjusted RR (95% CI)	*p*-Value
EU(7)-PIM list	Total medicines per patient	1.06 (1.06–1.07)	<0.001
	Total diagnoses per patient	0.98 (0.975–1.00)	0.0065
2019 AGS Beers criteria	Total medicines per patient	1.05 (1.05–1.06)	<0.001
	Total diagnoses per patient	0.99 (0.98–1.00)	0.0053
	E00-E89, endocrine, nutritional, and metabolic diseases	0.96 (0.92–1.00)	0.0382
	F01-F99, mental, behavioral, and neurodevelopmental disorders	1.12 (1.01–1.23)	0.0283
	I00-I99, diseases of the circulatory system	1.08 (1.05–1.12)	<0.001
STOPP v2 criteria	Total medicines per patient	1.06 (1.05–1.07)	<0.001
	Total diagnoses per patient	0.98 (0.97–1.00)	0.017
	I00-I99, diseases of the circulatory system	1.05 (1–1.09)	0.0477
	M00-M99, diseases of the musculoskeletal system and connective tissue	0.82 (0.67–1.00)	0.0491
EU(7)-PIM list	Total medicines per patient	1.064 (1.0575–1.070)	<0.001
	Total diagnoses per patient	0.983 (0.9715–0.995)	0.0065
2019 AGS Beers criteria	Total medicines per patient	1.054 (1.0495–1.059)	<0.001
	Total diagnoses per patient	0.986 (0.9765–0.996)	0.0053
STOPP v2 criteria	Total medicines per patient	1.063 (1.0555–1.07)	<0.001
	Total diagnoses per patient	0.984 (0.9715–0.997)	0.017

## Discussion

According to our knowledge, this is the first study assessing the concordance and overlap of three distinct PIM-detecting tools EU(7)-PIM list, 2019 AGS Beers criteria, and STOPP v2 criteria in hospitalized patients. The low overlap and concordance between tools highlight the need to develop a PIM-detecting tool for patients exposed to a high number of PIMs (≈80%, in all tools used) and reinforce the fact that general internal medicine patients are at risk of PIM (Hudhra et al., 2016; Blanc et al., 2018a; Blanc et al., 2018b). Although being developed for different drug markets and different populations, these criteria are the most used. For this reason, analyzing the concordance among tools is essential to understand the applicability of each tool in a specific population, country, and setting. Because multiple comorbidities are frequent among internal medicine inpatients, a tool focusing on geriatric internal medicine patients should be implemented to alert the physician to an eventual PIM prescription.

Despite the scarcity of studies comparing the use of PIM tools in all settings and the lack of studies in internal medicine inpatients, a study carried out in Chinese hospitalized patients reported a moderate concordance between 2015 Beers criteria and STOPP v2 criteria (Ma et al., 2019). Moreover, a Brazilian study performed in home-dwelling population of 60 or more years of age concluded that there was a high concordance among 2015 Beers criteria, STOPP v2 criteria, and the EU(7)-PIM list (Novaes et al., 2017). However, in a recent systematic review, a substantial difference was found between the individual medications identified by the Beers and STOPP/START criteria, highlighting the need for research in this area (Thomas and Thomas, 2019). The poor concordance among criteria observed in our sample of Portuguese internal medicine inpatients can be due to the applicability requirements of each list; theoretically, criteria with fewer applicability requirements might detect fewer PIMs than those using criteria that require more specific information, differential medication availability between countries (Chang and Chan, 2010; Thomas and Thomas, 2019). According to the EU(7)-PIM list, to consider the medicine as a PIM, it is only necessary to know the mediation profile of the patients including the duration of treatment and dosage of some medicine (Renom-Guiteras et al., 2015). The Beers criteria judge each medicine as a PIM based not only on the medication profile of a patient but also on the pathologies of the patients as well as the laboratory results (By the American Geriatrics Society Beers Criteria Update Expert, 2019). To apply the STOPP criteria, it is imperative to know the entire medication history, clinical information of the patient, and laboratory (O’mahony et al., 2015; By the American Geriatrics Society Beers Criteria Update Expert, 2019; Carvalho et al., 2019). The greater sensibility of previous versions of STOPP criteria was demonstrated by others (Gallagher and O’mahony, 2008; Hamilton et al., 2011; Wickop et al., 2016), but according to Blanco-Reina et al. (2019), STOPP v2 has a poor concordance with the previous version (Blanco-Reina et al., 2019).

The number of PIMs identified varies among criteria, and in the inpatient setting, the prevalence of PIM changes from 1% to as high as 50% and is highly dependent on the tool used to define PIM (Franceschi et al., 2008; Rothberg et al., 2008; Page et al., 2010). A study carried out in Portuguese nursing homes and day-care centers detected a PIM prevalence of 64.4% when applying the EU(7)-PIM list, 56% when applying the 2015 Beers criteria, and 85.5% when applying the STOPP v2 criteria (Monteiro et al., 2020). Another study carried out in Chinese inpatients reported a prevalence of PIM of 58.1 and 44.0% using 2015 Beers criteria and 2014 STOPP (Ma et al., 2019). A Brazilian study performed in a home-dwelling population of sixty or more years of age observed a prevalence of PIM of 50, 46.2, and 59.5% using, respectively, 2015 Beers criteria, 2015 STOPP criteria, and the EU(7)-PIM list (Novaes et al., 2017). In our study, the percentage of patients with at least one PIM also varied among criteria: according to the EU(7)-PIM list and STOPP criteria, near 80% of the patients had at least one PIM, and according to Beers criteria, more than 90% of the patients consume one PIM. Another study in patients discharged from a hospital using the EU(7)-PIM list and the STOPP criteria observed a prevalence of PIM similar to that observed in our study (Mucalo et al., 2017).

The overlap of three criteria revealed that the drugs that act on the nervous system are the most common, making a total of 20, and haloperidol is the most frequent PIM. Haloperidol is an antipsychotic drug that can help relieve disorders such as delusions or hallucinations in schizophrenic situations, but it can also be used in older patients with agitation or aggression, which thus may explain the high consumption of this medication in the study population (Potter et al., 2006). Several studies report that delirium is associated with substantial rates of morbidity and mortality in inpatients, which becomes a growing problem due to increased life expectancy. Haloperidol is currently the drug of choice for the treatment of delirium (Schrijver et al., 2014; Ostinelli et al., 2017; Herling et al., 2018a; Herling et al., 2018b).

The knowledge of the pharmacotherapeutic profile of each patient allowed the application of the EU(7)-PIM list and the identification of 63-PIM–related medicines, performing a total of 1,146 PIMs. These observations allowed concluding that the inpatients included in this study consume a high number of PIMs in comparison with other studies using this tool in European older inpatients (Mucalo et al., 2017; Bobrova et al., 2019; Wamil et al., 2019).

According to Beers criteria, our patients presented 1,829 PIMs related to the prescription of 77 different medicines. According to our knowledge, this is the first study that uses the AGS 2019 Beers criteria with inpatients. However, in comparison with studies using 2015 Beers criteria, our sample presented a very high prevalence of PIM (Juliano et al., 2018; Thomas et al., 2020; Zhang et al., 2020).

A Portuguese study reported that the STOPP/START criteria are useful tools to perform medication review in nursing home patients and changes of drug therapy because besides detecting PIM, they also allowed the detection of DRPs related to the non-drug treatment despite existing indication (Silva et al., 2014). The application of STOPP criteria allowed concluding that, according to these criteria, the number of PIMs prescribed to older inpatients follows that observed in studies from Canada (Thomas et al., 2020) and Spain (Martin et al., 2017) but is very high when compared with the number of PIMs observed in Malaysia (Loganathan et al., 2019) and Swiss (Urfer et al., 2016).

In our sample of older inpatients, it was observed that the number of PIMs per patient increases with the increased number of prescribed medicines and the time of hospitalization. According to Wickop et al. (2016), the number of medicines has a significant effect on the amount of PIMs detected. (Wickop et al., 2016).

The mean age of the included participants reflects the high life expectancy observed in Portugal (INE, 2017; PORDATA, 2020). The high number of prescribed medicines is probably due to the multiple comorbidities presented by the inpatients. According to the literature, the inpatient setting may predispose older adults to new prescriptions and probably unnecessary drugs (Page et al., 2010). In an acute care setting, it is difficult to convince physicians to change or discontinue chronic medication, particularly if the medication is not related to the reason for hospitalization (Page et al., 2010). Moreover, we observed a trend of increased polypharmacy with the length of stay in the hospital. Despite the scarcity of studies characterizing the medication profile of internal medicine inpatients, a study pointed out that the mean of prescribed drugs increases from 5.6 (at hospital admission) to 7.6 (at discharge) (Vonbach et al., 2008). Other studies demonstrate that the number of regular medicines in hospitalized older patients is high, and according to Connor et al. (2020), the median number can range from 11 (IGR 8 to 15) (at hospital admission) to 9 (at discharge). According to Hubbard et al. (2015), the mean number of regular medicines per day ranges from 7.1 to 7.6 at admission and discharge, respectively.

This study demonstrated that the number of medicines is associated with the use of PIM detected by the EU(7)-PIM list and Beers and STOPP criteria; indeed, polypharmacy is associated with the use of PIM in older adults (Steinman et al., 2006).

Although the consensus-based lists of medications, such as the EU(7)-PIM list, Beers criteria, and STOPP criteria, were valuable tools to detect PIM in older adults, the data of this study only represent the patients that have been admitted during 2019 to the internal medicine ward; for these reasons (specific setting and the limited number of samples), they cannot be generalized to the whole hospital population (Tanaka et al., 2015). Moreover, potential ADRs associated with PIM prescriptions were not evaluated because the hospital's electronic medical record used did not include information regarding ADRs.

However, the information collected in this study reinforces the need to optimize criteria adapted to the internal medicine and implement strategies that support the physician’s decision when prescribing a possible PIM but always leaving the possibility of judgment and medical decision. Adaptation of these tools to a consensus tool for specific condition was already done for the management of pain and inflammation in older adults (Motter et al., 2019).

The high number of PIMs observed during this study highlights the need for interventional studies to improve medication appropriateness among hospitalized older patients (Thomas and Thomas, 2019), particularly in internal medicine wards where there is a frequent need to change medication to achieve stabilization of patients. The increased risk of polypharmacy-related ADR (Schmiedl et al., 2018) in older patients demonstrates the need for clinical practice guidelines in polymedicated older patients and the development of educational interventions to promote and improve the use of PIM tools by healthcare professionals.

## Data Availability

All data generated or analysed during this study are included in the article/Supplementary Material, and further inquiries can be directed to the corresponding author.
