# Supplementary material for: Prescription of Potentially Inappropriate Medication in Older Inpatients of an Internal Medicine Ward: Concordance and Overlap Among the EU(7)-PIM List and Beers and STOPP Criteria
Source: Front Pharmacol. 2021 Jul 30;12:676020. doi: 10.3389/fphar.2021.676020 (PMC8362883; doi:10.3389/fphar.2021.676020)
Supplement: Supplementary file 1 [file Table1.DOCX]

Table S1 – Total Number of PIM prescribed, according to EU(7)-PIM list

| ATC  Code | Potentially inappropriate medications from the EU(7)-PIM List | PIM Frequency  N = 1146  (mean PIM/elderly=1.9) | % PIM |
| --- | --- | --- | --- |
| A02BA02 | Ranitidine | 9 | 0.8% |
| A02BC | Proton pump inhibitors | 2 | 0.2% |
| A03FA01 | **Metoclopramide** | **192** | **16.8%** |
| A03FA03 | Domperidone | 0 | - |
| A06AB02 | **Bisacodyl** | **110** | **9.6%** |
| A07DA03 | Loperamide (> 2 days) | 9 | 0.8% |
| A10A | Insulin – Only if used in "*sliding scale*" therapeutic scheme | No information | - |
| A10BH01 | Sitagliptin | 49 | 4.3% |
| B01AE07 | Dabigatran etexilate | 1 | 0.1% |
| B01AF01 | Rivaroxaban | 7 | 0.6% |
| B03AA | Iron bivalent, oral preparations | 41 | 3.6% |
| C01AA05 | Digoxin | 57 | 5.0% |
| C01AA08 | Metildigoxin | 23 | 2.0% |
| C01BC03 | Propafenone | 1 | 0.1% |
| C01BD01 | Amiodarone | 44 | 3.8% |
| C01EB17 | Ivabradine | 1 | 0.1% |
| C02AC01 | Clonidine | 5 | 0.4% |
| C02AC06 | Rilmenidine | 25 | 2.2% |
| C03DA01 | Spironolactone (> 25 mg/day) | 23 | 2.0% |
| C04AD03 | Pentoxifylline | 3 | 0.3% |
| C07AA05 | Propranolol | 9 | 0.8% |
| C07AA07 | Sotalol | 2 | 0.2% |
| C07AG01 | Labetalol | 10 | 0.9% |
| C08CA05 | Nifedipine | 10 | 0.9% |
| G04BD02 | Flavoxate | 2 | 0.2% |
| J01XE01 | Nitrofurantoin (> 1 week) | 2 | 0.2% |
| M01AB05 | Diclofenac | 6 | 0.5% |
| M01AE02 | Naproxen (>2 x 250 mg/day or for a period longer than a week) | 10 | 0.9% |
| M03BX01 | Baclofen | 6 | 0.5% |
| M04AC01 | Colchicine | 15 | 1.3% |
| N02AX02 | Tramadol | 36 | 3.1% |
| N02BA01 | Acetylsalicylic acid (> 325 mg) | 5 | 0.4% |
| N03AA02 | Phenobarbital | 2 | 0.2% |
| N03AB02 | Phenytoin | 1 | 0.1% |
| N03AE01 | Clonazepam | 10 | 0.9% |
| N03AF01 | Carbamazepine | 7 | 0.6% |
| N03AX11 | Topiramate | 3 | 0.3% |
| N04AA02 | Biperiden | 5 | 0.4% |
| N04BD01 | Selegilline | 2 | 0.2% |
| N05AA01 | Chlorpromazine | 21 | 1.8% |
| N05AA02 | Levomepromazine | 2 | 0.2% |
| N05AA06 | Cyamemazine | 1 | 0.1% |
| N05AD01 | **Haloperidol (> 2mg at a single dose; >5 mg/day)** | **143** | **12.5%** |
| N05AH03 | Olanzapine (> 10 mg/day) | 0 | - |
| N05AN01 | Lithium | 1 | 0.1% |
| N05AX08 | Risperidone (> 6 weeks) | 0 | - |
| N05BA01 | Diazepam | 43 | 3.8% |
| N05BA04 | Oxazepam (> 60 mg/day) | 0 | - |
| N05BA06 | Lorazepam (>1 mg/day) | 31 | 2.7% |
| N05BA08 | Bromazepam | 9 | 0.8% |
| N05BA12 | Alprazolam | 58 | 5.1% |
| N05BA18 | Ethyl loflazepate | 2 | 0.2% |
| N05BB01 | Hydroxyzine | 50 | 4.4% |
| N05CD08 | Midazolam | 5 | 0.4% |
| N05CF02 | Zolpidem (> 5mg/day) | 0 | - |
| N06AA04 | Clomipramine | 2 | 0.2% |
| N06AA09 | Amitriptyline | 9 | 0.8% |
| N06AB03 | Fluoxetine | 7 | 0.6% |
| N06AB05 | Paroxetine | 3 | 0.3% |
| N06AX12 | Bupropion | 1 | 0.1% |
| N06AX16 | Venlafaxine | 5 | 0.4% |
| R06AA04 | Clemastine | 8 | 0.7% |

Table S2– Total number of PIM PRESCRIBED, ACCORDING TO BEERS CRITERIA

| ATC  Code | Total number of Potentially inappropriate medications according to 2019 Beers Criteria | PIM Frequency  N = 1829  (mean PIM/elderly=3) | % PIM |  |  |
| --- | --- | --- | --- | --- | --- |
| A02BA02 | Ranitidine | 0 | - |  |  |
| A02BC | Proton pump inhibitors | 2 | 0.1% |  |  |
| A03BB01 | Butylescopolamine | 14 | 0.8% |  |  |
| A03FA01 | **Metoclopramide** | **192** | **10.5%** |  |  |
| A07EA06 | Budesonide | 1 | 0.1% |  |  |
| A10A | Insulin – Only if used in "*sliding scale*" therapeutic scheme | (no information) | - |  |  |
| B01AB05 | Enoxaparin | 4 | 0.2% |  |  |
| B01AC06 | Acetylsalicylic acid | 72 | 3.9% |  |  |
| B01AE07 | Dabigatran etexilate | 0 | - |  |  |
| B01AF01 | Rivaroxaban | 0 | - |  |  |
| B01AF03 | Edoxaban | 0 | - |  |  |
| C01AA05 | Digoxin (First-line treatment of atrial fibrillation or heart failure) | 44 | 2.4% |  |  |
| C01BD01 | Amiodarone | 44 | 2.4% |  |  |
| C02AC01 | Clonidine (First-line hypertension treatment) | 0 | - |  |  |
| C03BA08 | Metolazone | 24 | 1.3% |  |  |
| C03CA01 | **Furosemide** | **437** | 23.9% |  |  |
| C03DA01 | Spironolactone | 107 | 5.9% |  |  |
| C08CA05 | Nifedipine (immediate release) | 7 | 0.4% |  |  |
| G04BD02 | Flavoxate | 0 | - |  |  |
| H01BA02 | Desmopressin | 1 | 0.1% |  |  |
| H02AB02 | Dexamethasone | 0 | - |  |  |
| H02AB04 | Methylprednisolone | 0 | - |  |  |
| H02AB06 | Prednisolone | 0 | - |  |  |
| H02AB09 | Hydrocortisone | 0 | - |  |  |
| H02AB13 | Deflazacort | 1 | 0.1% |  |  |
| J01EE01 | Sulfamethoxazole and trimethoprim | 24 | 1.3% |  |  |
| J01MA02 | Ciprofloxacin | 0 | - |  |  |
| J01XE01 | Nitrofurantoin | 6 | 0.3% |  |  |
| M01AB05 | Diclofenac | 6 | 0.3% |  |  |
| M01AE02 | Naproxen | 33 | 1.8% |  |  |
| M01AH04 | Parecoxib | 0 | - |  |  |
| N02AA01 | Morphine | 1 | 0.1% |  |  |
| N02AB03 | Fentanyl | 0 | - |  |  |
| N02AX02 | Tramadol | 11 | 0.6% |  |  |
| N02AX06 | Tapentadol | 2 | 0.1% |  |  |
| N02BA01 | Acetylsalicylic acid (> 325 mg/day) | 5 | 0.3% |  |  |
| N03AA02 | Phenobarbital | 2 | 0.1% |  |  |
| N03AE01 | Clonazepam | 10 | 0.5% |  |  |
| N03AF01 | Carbamazepine | 7 | 0.4% |  |  |
| N03AX12 | Gabapentin | 0 | - |  |  |
| N03AX14 | Levetiracetam | 0 | - |  |  |
| N03AX16 | Pregabalin | 0 | - |  |  |
| N05AA01 | Chlorpromazine | 21 | 1.1% |  |  |
| N05AA02 | Levomepromazine | 2 | 0.1% |  |  |
| N05AA06 | Cyamemazine | 1 | 0.1% |  |  |
| N05AD01 | **Haloperidol** | **148** | 8.1% |  |  |
| N05AD03 | Melperone | 22 | 1.2% |  |  |
| N05AH03 | Olanzapine | 6 | 0.3% |  |  |
| N05AH04 | Quetiapine | 88 | 4.8% |  |  |
| N05AL01 | Sulpiride | 1 | 0.1% |  |  |
| N05AL03 | Tiapride | 14 | 0.8% |  |  |
| N05AL05 | Amisulpride | 3 | 0.2% |  |  |
| N05AX08 | Risperidone | 12 | 0.7% |  |  |
| N05AX13 | Paliperidone | 1 | 0.1% |  |  |
| N05BA | Mexazolam | 1 | 0.1% |  |  |
| N05BA01 | Diazepam | 43 | 2.4% |  |  |
| N05BA04 | Oxazepam | 65 | 3.6% |  |  |
| N05BA06 | Lorazepam | 69 | 3.8% |  |  |
| N05BA08 | Bromazepam | 1 | 0.1% |  |  |
| N05BA12 | Alprazolam | 58 | 3.2% |  |  |
| N05BA18 | Ethyl loflazepate | 0 | - |  |  |
| N05BB01 | Hydroxyzine | 50 | 2.7% |  |  |
| N05CD08 | Midazolam | 2 | 0.1% |  |  |
| N05CF02 | Zolpidem | 2 | 0.1% |  |  |
| N06AA04 | Clomipramine | 2 | 0.1% |  |  |
| N06AA09 | Amitriptyline | 9 | 0.5% |  |  |
| N06AB03 | Fluoxetine | 7 | 0.4% |  |  |
| N06AB05 | Paroxetine | 3 | 0.2% |  |  |
| N06AB06 | Sertraline | 38 | 2.1% |  |  |
| N06AB10 | Escitalopram | 30 | 1.6% |  |  |
| N06AX05 | Trazodone | 31 | 1.7% |  |  |
| N06AX11 | Mirtazapine | 27 | 1.5% |  |  |
| N06AX16 | Venlafaxine | 5 | 0.3% |  |  |
| N06AX21 | Duloxetine | 2 | 0.1% |  |  |
| N06DA02 | Donepezil | 0 | - |  |  |
| R06AA04 | Clemastine | 8 | 0.4% |  |  |

#### Table S3- Potentially inappropriate medications identified through the application of the table 2 of beers criteria- PIM in the elderly regardless of the clinical situation.

| ATC code | Potentially Inappropriate Medication in Older Patients apart from the Clinical Condition | Frequency  N=979 |
| --- | --- | --- |
| A02BC | Proton pump inhibitors | 2 |
| A03FA01 | **Metoclopramide** | **192** |
| A10A | Insulin – Only if used in "*sliding scale*" therapeutic scheme | No information |
| C01AA05 | Digoxin | 44 |
| C01BD01 | Amiodarone | 44 |
| C02AC01 | Clonidine | 0 |
| C08CA05 | Nifedipine (immediate release) | 7 |
| H01BA02 | Desmopressin | 1 |
| J01XE01 | Nitrofurantoin | 6 |
| M01AB05 | Diclofenac | 6 |
| M01AE02 | Naproxen | 33 |
| N02BA01 | Acetylsalicylic acid (> 325 mg) | 5 |
| N03AA02 | Phenobarbital | 2 |
| N03AE01 | Clonazepam | 10 |
| N05AA01 | Chlorpromazine | 21 |
| N05AA02 | Levomepromazine | 2 |
| N05AA06 | Cyamemazine | 1 |
| N05AD01 | Haloperidol | 148 |
| N05AD03 | Melperone | 22 |
| N05AH03 | Olanzapine | 6 |
| N05AH04 | Quetiapine | 88 |
| N05AL01 | Sulpiride | 1 |
| N05AL03 | Tiapride | 14 |
| N05AL05 | Amisulpride | 3 |
| N05AX08 | Risperidone | 12 |
| N05AX13 | Paliperidone | 1 |
| N05BA01 | Diazepam | 43 |
| N05BA04 | Oxazepam | 65 |
| N05BA06 | Lorazepam | 69 |
| N05BA08 | Bromazepam | 1 |
| N05BA12 | Alprazolam | 58 |
| N05BB01 | Hydroxyzine | 50 |
| N05CF02 | Zolpidem | 2 |
| N06AA09 | Amitriptyline | 9 |
| N06AB05 | Paroxetine | 3 |
| R06AA04 | Clemastine | 8 |

Table S4- Potentially inappropriate medications identified through the application of the table 3 of beers criteria- PIM in the elderly considering their clinical situation.

| Diagnose | ATC code | Potentially Inappropriate Medications | Frequency  N=221 |
| --- | --- | --- | --- |
| Heart failure | M01AB05 | Diclofenac | 1 |
|  | M01AE02 | Naproxen | 8 |
|  | N05AA01 | Chlorpromazine | 6 |
|  | N05AH03 | Olanzapine | 1 |
|  | N06AA04 | Clomipramine | 1 |
|  | N06AA09 | Amitriptyline | 4 |
|  | N06AX05 | Trazodone | 30 |
|  | N06AX11 | Mirtazapine | 6 |
|  | N06DA02 | Donepezil | 2 |
|  | **Total** |  | **59 (26.7%)** |
| Delirium | N05BA01 | Diazepam | 1 |
|  | N05BA06 | Lorazepam | 1 |
|  | **Total** |  | **2 (0.9%)** |
| Dementia or cognitive impairment | A03BB01 | Butylscopolamine | 9 |
|  | A07EA06 | Budesonide | 1 |
|  | H02AB13 | Deflazacort | 1 |
|  | N05AA01 | Chlorpromazine | 2 |
|  | N05AD01 | Haloperidol | 21 |
|  | N05AD03 | Melperone | 8 |
|  | N05AH03 | Olanzapine | 4 |
|  | N05AH04 | Quetiapine | 19 |
|  | N05AL03 | Tiapride | 1 |
|  | N05AL05 | Amisulpride | 1 |
|  | N05AX08 | Risperidone | 4 |
|  | N05BA | Mexazolam | 1 |
|  | N05BA01 | Diazepam | 6 |
|  | N05BA04 | Oxazepam | 6 |
|  | N05BA06 | Lorazepam | 10 |
|  | N05BA08 | Bromazepam | 1 |
|  | N05BA12 | Alprazolam | 5 |
|  | N05BB01 | Hydroxyzine | 4 |
|  | N05CD08 | Midazolam | 2 |
|  | N06AA04 | Clomipramine | 1 |
|  | R06AA04 | Clemastine | 1 |
|  | **Total** |  | **108 (48.9%)** |
| History of falls or  fractures | N02AA01 | Morphine | 1 |
|  | N02AX02 | Tramadol | 1 |
|  | N02AX06 | Tapentadol | 2 |
|  | N05BA | Mexazolam | 1 |
|  | N05BA01 | Diazepam | 4 |
|  | N05BA04 | Oxazepam | 2 |
|  | N05BA06 | Lorazepam | 3 |
|  | N05BA12 | Alprazolam | 1 |
|  | N06AX05 | Trazodone | 2 |
|  | N06AX11 | Mirtazapine | 2 |
|  | **Total** |  | **19 (8.6%)** |
| Parkinson disease | A03FA01 | Metoclopramide | 6 |
|  | N05AA01 | Chlorpromazine | 1 |
|  | N05AD01 | Haloperidol | 3 |
|  | **Total** |  | **10 (4.5%)** |
| Gastrointestinal  History of gastric or duodenal ulcers | M01AE02 | Naproxen | 1 |
|  | **Total** |  | **1 (0.5%)** |
| Lower urinary tract symptoms, benign prostatic hyperplasia | A03BB01 | Butylscopolamine | 4 |
|  | N05AA01 | Chlorpromazine | 5 |
|  | N05AH03 | Olanzapine | 3 |
|  | N05BB01 | Hydroxyzine | 5 |
|  | N06AA04 | Clomipramine | 1 |
|  | N06AA09 | Amitriptylina | 1 |
|  | R06AA04 | Clemastine | 3 |
|  | **Total** |  | **22 (10%)** |

Table S5- Potentially inappropriate medications identified through the application of the table 4 of beers criteria- drugs to be used with caution in older adults.

| ATC code | Potentially Inappropriate Medications: Drugs To Be Used With Caution in Older Adults | Frequency  N=1226 |
| --- | --- | --- |
| B01AC06 | Acetylsalicylic acid | 72 |
| B01AE07 | Dabigatran | 0 |
| B01AF01 | Rivaroxaban | 0 |
| C03BA08 | Metolazone | 24 |
| C03CA01 | **Furosemide** | **437** |
| C03DA01 | Spironolactone | 107 |
| J01EE01 | sulfamethoxazole and trimethoprim | 24 |
| N02AX02 | Tramadol | 36 |
| N03AF01 | Carbamazepine | 7 |
| N05AA01 | Chloopromazine | 21 |
| N05AA02 | Levomepromazine | 2 |
| N05AA06 | Cyamemazine | 1 |
| N05AD01 | **Haloperidol** | **148** |
| N05AD03 | Melperone | 22 |
| N05AH03 | Olanzapine | 6 |
| N05AH04 | **Quetiapine** | **88** |
| N05AL01 | Sulpiride | 1 |
| N05AL03 | Tiapride | 14 |
| N05AL05 | Amisulpride | 3 |
| N05AX08 | Risperidone | 12 |
| N05AX13 | Paliperidone | 1 |
| N06AA04 | Clomipramine | 2 |
| N06AA09 | Amitriptyline | 9 |
| N06AB03 | Fluoxetine | 7 |
| N06AB05 | Paroxetine | 3 |
| N06AB06 | Sertraline | 38 |
| N06AB10 | Escitalopram | 30 |
| N06AX05 | Trazadone | 77 |
| N06AX11 | Mirtazapine | 27 |
| N06AX16 | Venlafaxine | 5 |
| N06AX21 | Duloxetine | 2 |

Table S6- Potentially Clinically Important Drug-Drug Interactions That Should Be Avoided in Older Adults, identified through the application of table 5 of Beers criteria

| Interacting Drug and Class | Object Drug and Class | | Frequency  N = 263 |
| --- | --- | --- | --- |
| ACEIs, ARBs | Spironolactone, Enalapril, Lisinopril, Perindopril, Ramipril, Losartan , Valsartan, Candesartan | | **68** |
| Benzodiazepines | Morphine, Fentanyl, Tramadol, Tapentadol | | **41** |
| Gabapentin, pregabalin | Morphine, Fentanyl, Tramadol, Tapentadol | | **12** |
| Anticholinergic | Butylscopolamine, Flavoxate, Chlorpromazine, Olanzapine, Hydroxyzine, Clomipramine, Amitriptylina, Paroxetine, Clemastine | | **8** |
| NSAIDs | Budesonide, Dexametasona, Metilprednisolona, Prednisolona, Hidrocortisona, Deflazacorte | | **8** |
| Any combination of three  or more of these  CNS-active drugs | Morphine, Fentanyl, Tramadol, Tapentadol, Phenobarbital, Phenytoin, Clonazepam, Carbamazepine, Valproic acid, Topiramate, Gabapentin, Levetiracetam, Pregabalin, Chlorpromazine, Levomepromazine, Cyamemazine, Haloperidol, Melperone, Olanzapine, Quetiapine, Sulpiride, Tiapride, Amisulpride, Risperidone, Paliperidone, Mexazolam, Diazepam, Oxazepam, Lorazepam, Bromazepam, Alprazolam, Ethyl loflazepate, Midazolam, Zolpidem, Clomipramine, Amitriptyline, Fluoxetine, Paroxetine, Setraline, Escitalopram, Trazodone, Mirtazapine, Venlafaxine, Duloxetine | | **125** |
| ACEIs | N05AN01 | Lithium | 0 |
| Loop diuretics | N05AN01 | Lithium | 0 |
| Trimethoprim-sulfamethoxazole | N03AB02 | Phenitoin | 0 |
| Amiodarone | B01AA03 | Warfarin | **1** |
| Ciprofloxacin | B01AA03 | Warfarin | 0 |
| Macrolides (excluding  azithromycin) | B01AA03 | Warfarin | 0 |
| Trimethoprim-sulfamethoxazole | B01AA03 | Warfarin | 0 |
| NSAIDs | B01AA03 | Warfarin | 0 |

Table S7- Medications that should be avoided or have their dosage reduced with varying levels of kidney function in older adults, identified through the application of table 6 of Beers criteria

| ATC code | Medications That Should Be Avoided or Have Their Dosage Reduced with Varying Levels of Kidney Function in Older Adults | Frequency  N = 6 |
| --- | --- | --- |
| B01AB05 | Enoxaparin | 4 |
| B01AE07 | Dabigatran | 0 |
| B01AF01 | Rivaroxaban | 0 |
| B01AF03 | Edoxaban | 0 |
| C03DA01 | Spironolactone | 1 |
| J01EE01 | Sulfamethoxazole and trimethoprim | 1 |
| J01MA02 | Ciprofloxacin | 0 |
| N02AX02 | Tramadol | 0 |
| N03AX12 | Gabapentin | 0 |
| N03AX14 | Levetiracetam | 0 |
| N03AX16 | Pregabalin | 0 |
| N06AX21 | Duloxetine | 0 |

Table S8- Drugs with strong anticholinergic properties, identified through the application of table 7 of Beers criteria

| ATC code | Drugs With Strong Anticholinergic Properties | Frequency  N=133 |
| --- | --- | --- |
| A03BB01 | Butylscopolamine | 32 |
| G04BD02 | Flavoxate | 2 |
| N05AA01 | Chlorpromazine | 21 |
| N05AH03 | Olanzapine | 6 |
| N05BB01 | Hydroxiyina | 50 |
| N06AA04 | Clomipramine | 2 |
| N06AA09 | Amitriptyline | 9 |
| N06AB05 | Paroxetine | 3 |
| R06AA04 | Clemastine | 8 |

Table S9- Total number of potentially inappropriate medications according to STOPP criteria

| ATC code | POTENTIALLY INAPPROPRIATE MEDICATIONS | PIM Frequency  N = 1156  (mean PIM/elderly=1.9) | % PIM |
| --- | --- | --- | --- |
| A02BC01 | Omeprazol | - | - |
| A02BC02 | Pantoprazol | - | - |
| A03BB01 | Butylescopolamine | 20 | 1.7% |
| A03FA01 | Metoclopramide | 13 | 1.1% |
| A10BA02 | Metformin | 0 | - |
| A14AB01 | Nandrolone | 2 | 0.2% |
| B01AA03 | Warfarin | - | - |
| B01AC04 | Clopidogrel | 3 | 0.3% |
| B01AC06 | Acetylsalicylic acid | 11 | 1.0% |
| B01AC18 | Triflusal | 0 | 0.0% |
| B01AC24 | Ticagrelor | 1 | 0.1% |
| B01AE07 | Dabigatran | - | - |
| B01AF01 | Rivaroxaban | - | - |
| B01AF03 | Edoxaban | - | - |
| B03AA07 | Ferrous sulfate | 9 | 0.8% |
| C01AA05 | Digoxin | - | - |
| C01BD01 | Amiodarone | - | - |
| C01DA02 | Glyceryl trinitrate | 3 | 0.3% |
| C01DA08 | Isosorbide dinitrate | 1 | 0.1% |
| C01DA14 | Isosorbide mononitrate | 2 | 0.2% |
| C02AB02 | Methyldopa | - | - |
| C02AC01 | Clonidine | - | - |
| C02AC06 | Rilmenidine | - | - |
| C03BA04 | Chlortalidone | 0 | - |
| C03BA08 | Metolazone | 6 | 0.5% |
| C03BA11 | Indapamide | 0 | - |
| C03CA01 | Furosemide | 39 | 3.4% |
| C03DA01 | **Spironolactone** | **79** | **6.8%** |
| C07AA05 | Propranolol | 3 | 0.3% |
| C07AA07 | Sotalol | 1 | 0.1% |
| C07AB02 | Metoprolol | 0 | - |
| C07AB07 | Bisoprolol | 33 | 2.9% |
| C07AB12 | Nebivolol | 1 | 0.1% |
| C07AG02 | Carvedilol | 1 | 0.1% |
| C08CA01 | Amlodipine | 7 | 0.6% |
| C08CA05 | Nifedipine | 2 | 0.2% |
| C08CA06 | Nimodipine | 0 | - |
| C09AA01 | Captopril | 51 | 4.4% |
| C09AA02 | Enalapril | 3 | 0.3% |
| C09AA03 | Lisinopril | 1 | 0.1% |
| C09AA04 | Perindopril | 6 | 0.5% |
| C09AA05 | Ramipril | 40 | 3.5% |
| C09CA01 | Losartan | 1 | 0.1% |
| C09CA03 | Valsartan | 6 | 0.5% |
| C09CA06 | Candesartan | 1 | 0.1% |
| G04BD02 | Flavoxate | 2 | 0.2% |
| H02AB04 | Methylprednisolone | 10 | 0.9% |
| H02AB06 | Prednisolone | 4 | 0.3% |
| H02AB09 | Hydrocortisone | 7 | 0.6% |
| H02AB13 | Deflazacort | 4 | 0.3% |
| M01AB05 | Diclofenac | 2 | 0.2% |
| M01AE02 | Naproxen | 14 | 1.2% |
| M01AH04 | Parecoxib | 1 | 0.1% |
| M04AC01 | Colchicine | - | - |
| M05BA08 | Zoledronic acid | 0 | - |
| N02AA01 | Morphine | 34 | 2.9% |
| N02AB03 | Fentanyl | 8 | 0.7% |
| N02AX02 | Tramadol | 16 | 1.4% |
| N02AX06 | Tapentadol | 8 | 0.7% |
| N04AA02 | Biperiden | 0 | - |
| N05AA01 | Chlorpromazine | 21 | 1.8% |
| N05AA02 | Levomepromazine | 2 | 0.2% |
| N05AA06 | Cyamemazine | 1 | 0.1% |
| N05AD01 | **Haloperidol** | **148** | **12.8%** |
| N05AD03 | Melperone | 22 | 1.9% |
| N05AH03 | Olanzapine | 6 | 0.5% |
| N05AH04 | **Quetiapine** | **88** | **7.6%** |
| N05AL01 | Sulpiride | 1 | 0.1% |
| N05AL03 | Tiapride | 14 | 1.2% |
| N05AL05 | Amisulpride | 3 | 0.3% |
| N05AX08 | Risperidone | 12 | 1.0% |
| N05AX13 | Paliperidone | 1 | 0.1% |
| N05BA | Mexazolam | 2 | 0.2% |
| N05BA01 | Diazepam | 43 | 3.7% |
| N05BA04 | Oxazepam | 65 | 5.6% |
| N05BA06 | Lorazepam | 69 | 6% |
| N05BA08 | Bromazepam | 9 | 0.8% |
| N05BA12 | Alprazolam | 58 | 5.0% |
| N05BA18 | Ethyl loflazepate | 2 | 0.2% |
| N05BB01 | Hydroxyzine | 50 | 4.3% |
| N05CD08 | Midazolam | 5 | 0.4% |
| N05CF02 | Zolpidem | 2 | 0.2% |
| N06AA04 | Clomipramine | 1 | 0.1% |
| N06AA09 | Amitriptiline | 3 | 0.3% |
| N06AB03 | Fluoxetine | 0 | - |
| N06AB05 | Paroxetine | 3 | 0.3% |
| N06AB06 | Sertraline | 3 | 0.3% |
| N06AB10 | Escitalopram | 7 | 0.6% |
| N06AX05 | Trazodone | 33 | 2.9% |
| N06AX11 | Mirtazapine | 8 | 0.7% |
| N06DA02 | Donepezil | 5 | 0.4% |
| N07AA01 | Neostigmine | 0 | 0.0% |
| R03BB01 | Ipratropium bromide | 5 | 0.4% |
| R03BB04 | Tiotropium bromide | 0 | - |
| R06AA04 | Clemastine | 8 | 0.7% |

TabLe S10 - Screening Tool of Older Persons’ Prescriptions (STOPP) version 2

| Screening Tool of Older Persons’ Prescriptions (STOPP) version 2 | Frequency  N=1398 |
| --- | --- |
| Section A: Indication of medication | |
| Any drug prescribed without an evidence-based clinical indication. Any drug prescribed beyond the recommended duration, where treatment duration is well defined. Any duplicate drug class prescription e.g. two concurrent NSAIDs, SSRIs, loop diuretics, ACE inhibitors, anticoagulants (optimisation of monotherapy within a single drug class should be observed prior to considering a new agent). | **12**  **(0.9%)** |
| Section B: Cardiovascular System | |
| 4. Beta blocker with bradycardia (< 50/min), type II heart block or complete heart block (risk of complete heart block, asystole). | 34 |
| 7. Loop diuretic for dependent ankle oedema without clinical, biochemical evidence or radiological evidence of heart failure, liver failure, nephrotic syndrome or renal failure (leg elevation and /or compression hosiery usually more appropriate). | 34 |
| 8. Thiazide diuretic with current significant hypokalaemia (i.e. serum K+ < 3.0 mmol/l), hyponatraemia (i.e. serum Na+ < 130 mmol/l) hypercalcaemia (i.e. corrected serum calcium > 2.65 mmol/l) or with a history of gout (hypokalaemia, hyponatraemia, hypercalcaemia and gout can be precipitated by thiazide diuretic) | 6 |
| 9. Loop diuretic for treatment of hypertension with concurrent urinary incontinence (may exacerbate incontinence). | 5 |
| 11. ACE inhibitors or Angiotensin Receptor Blockers in patients with hyperkalaemia. | 106 |
| 12. Aldosterone antagonists (e.g. spironolactone, eplerenone) with concurrent potassium-conserving drugs (e.g. ACEI’s, ARB’s, amiloride, triamterene) without monitoring of serum potassium (risk of dangerous hyperkalaemia i.e. > 6.0 mmol/l – serum K should be monitored regularly, i.e. at least every 6 months). | 79 |
| Total | **264 (18.9%)** |
| Section C: Antiplatelet/Anticoagulant Drugs | |
| 1. Long-term aspirin at doses greater than 160mg per day (increased risk of bleeding, no evidence for increased efficacy). | 5 |
| 2. Aspirin with a past history of peptic ulcer disease without concomitant PPI (risk of recurrent peptic ulcer). | 2 |
| 5. Aspirin in combination with vitamin K antagonist, direct thrombin inhibitor or factor Xa inhibitors in patients with chronic atrial fibrillation (no added benefit from aspirin). | 3 |
| 6. Antiplatelet agents with vitamin K antagonist, direct thrombin inhibitor or factor Xa inhibitors in patients with stable coronary, cerebrovascular or peripheral arterial disease (No added benefit from dual therapy). | 5 |
| 10. NSAID and vitamin K antagonist, direct thrombin inhibitor or factor Xa inhibitors in combination (risk of major gastrointestinal bleeding). | 10 |
| 11. NSAID with concurrent antiplatelet agent(s) without PPI prophylaxis (increased risk of peptic ulcer disease) | 5 |
| Total | **30 (2.1%)** |
| Section D: Central Nervous System and Psychotropic Drugs | |
| 1. TriCyclic Antidepressants (TCAs) with dementia, narrow angle glaucoma, cardiac conduction abnormalities, prostatism, or prior history of urinary retention (risk of worsening these conditions). | 43 |
| 3. Neuroleptics with moderate-marked antimuscarinic/anticholinergic effects (chlorpromazine, clozapine, flupenthixol, fluphenzine, pipothiazine, promazine, zuclopenthixol) with a history of prostatism or previous urinary retention (high risk of urinary retention). | 0 |
| 4. Selective serotonin re-uptake inhibitors (SSRI’s) with current or recent significant hyponatraemia i.e. serum Na+ < 130 mmol/l (risk of exacerbating or precipitating hyponatraemia). | 13 |
| 6. Antipsychotics (i.e. other than quetiapine or clozapine) in those with parkinsonism or Lewy Body Disease (risk of severe extra-pyramidal symptoms) | 42 |
| 8. Anticholinergics/antimuscarinics in patients with delirium or dementia (risk of exacerbation of cognitive impairment). | 27 |
| 11. Acetylcholinesterase inhibitors with a known history of persistent bradycardia (< 60 beats/min.), heart block or recurrent unexplained syncope or concurrent treatment with drugs that reduce heart rate such as beta-blockers, digoxin, diltiazem, verapamil (risk of cardiac conduction failure, syncope and injury). | 5 |
| 14. First-generation antihistamines (safer, less toxic antihistamines now widely available). | 64 |
| Total | **194 (13.9%)** |
| Section F: Gastrointestinal System | |
| 1. Prochlorperazine or metoclopramide with Parkinsonism (risk of exacerbating Parkinsonian symptoms). | 13 |
| 3. Drugs likely to cause constipation (e.g. antimuscarinic/anticholinergic drugs, oral iron, opioids, verapamil, aluminium antacids) in patients with chronic constipation where non-constipating alternatives are available (risk of exacerbation of constipation). | 150 |
| 4. Oral elemental iron doses greater than 200 mg daily (e.g. ferrous fumarate> 600 mg/day, ferrous sulphate > 600 mg/day, ferrous gluconate> 1800 mg/day; no evidence of enhanced iron absorption above these doses). | 3 |
| Total | **166 (11.9%)** |
| Section G: Respiratory System | |
| 2. Systemic corticosteroids instead of inhaled corticosteroids for maintenance therapy in moderate-severe COPD (unnecessary exposure to long-term side-effects of systemic corticosteroids and effective inhaled therapies are available). | 20 |
| 3. Anti-muscarinic bronchodilators (e.g. ipratropium, tiotropium) with a history of narrow angle glaucoma (may exacerbate glaucoma) or bladder outflow obstruction (may cause urinary retention). | 4 |
| 4. Benzodiazepines with acute or chronic respiratory failure i.e. pO2 < 8.0 kPa ± pCO2 > 6.5 kPa (risk of exacerbation of respiratory failure). | 38 |
| Total | **62 (4.4%)** |
| Section H: Musculoskeletal System | |
| 1. Non-steroidal anti-inflammatory drug (NSAID) other than COX-2 selective agents with history of peptic ulcer disease or gastrointestinal bleeding, unless with concurrent PPI or H2 antagonist (risk of peptic ulcer relapse). | 1 |
| 5. Corticosteroids (other than periodic intra-articular injections for mono-articular pain) for osteoarthritis (risk of systemic corticosteroid side-effects). | 4 |
| 7. COX-2 selective NSAIDs with concurrent cardiovascular disease (increased risk of myocardial infarction and stroke) | 1 |
| 8. NSAID with concurrent corticosteroids without PPI prophylaxis (increased risk of peptic ulcer disease) | 1 |
| 9. Oral bisphosphonates in patients with a current or recent history of upper gastrointestinal disease i.e. dysphagia, oesophagitis, gastritis, duodenitis, or peptic ulcer disease, or upper gastrointestinal bleeding (risk of relapse/exacerbation of oesophagitis, oesophageal ulcer, oesophageal stricture) | 0 |
| Total | **7 (0.5%)** |
| Section I: Urogenital System | |
| 1. Antimuscarinic drugs with dementia, or chronic cognitive impairment (risk of increased confusion, agitation) or narrow-angle glaucoma (risk of acute exacerbation of glaucoma), or chronic prostatism (risk of urinary retention). | 0 |
| Section J. Endocrine System | |

| **3**. Beta-blockers in diabetes mellitus with frequent hypoglycaemic episodes (risk of suppressing hypoglycaemic symptoms). | 4 |
| --- | --- |
| **6.** Androgens (male sex hormones) in the absence of primary or secondary hypogonadism (risk of androgen toxicity; no proven benefit outside of the hypogonadism indication). | 2 |
| **Total** | **6 (0.4%)** |
| **Section K: Drugs that predictably increase the risk of falls in older people** | |
| **1.** Benzodiazepines (sedative, may cause reduced sensorium, impair balance). | 253 |
| **2.** Neuroleptic drugs (may cause gait dyspraxia, Parkinsonism). | 319 |
| **3.** Vasodilator drugs (e.g. alpha-1 receptor blockers, calcium channel blockers, long-acting nitrates, ACE inhibitors, angiotensin I receptor blockers,) with persistent postural hypotension i.e. recurrent drop in systolic blood pressure ≥ 20mmHg (risk of syncope, falls). | 17 |
| **4.** Hypnotic Z-drugs e.g. zopiclone, zolpidem, zaleplon (may cause protracted daytime sedation, ataxia). | 2 |
| **Total** | **591 (42.3%)** |
| **Section L: Analgesic Drugs** | |
| **2.** Use of regular (as distinct from PRN) opioids without concomitant laxative (risk of severe constipation). | **59 (4.2%)** |
| **Section N: Antimuscarinic/Anticholinergic Drug Burden** | |
| **1.** Concomitant use of two or more drugs with antimuscarinic/anticholinergic properties (e.g. bladder antispasmodics, intestinal antispasmodics, tricyclic antidepressants, first generation antihistamines) (risk of increased antimuscarinic/anticholinergic toxicity) | **7 (0.5%)** |
